# Hypovitaminosis D in the Greek Elderly Population Doesn't Concern Only Patients with Hip Fracture

**DOI:** 10.7759/cureus.9657

**Published:** 2020-08-11

**Authors:** Ioannis Papaioannou, Georgia Pantazidou, Ifigeneia Kostoglou-Athanasiou, Panagiotis Korovessis

**Affiliations:** 1 Orthopedics and Traumatology, General Hospital of Patras "Agios Andreas", Patras, GRC; 2 Otolaryngology-Head and Neck Surgery, General Hospital of Patras "Agios Andreas", Patras, GRC; 3 Endocrinology, Diabetes and Metabolism, General Hospital "Asklepieio Voulas", Athens, GRC

**Keywords:** elderly, hip fracture, osteoarthritis, complications, sunshine, vitamin d

## Abstract

Introduction

Vitamin D (VD) deficiency seems to be an underestimated public health issue, especially in countries with a significant duration of sunlight throughout the year, as in this sunny Mediterranean region where this cross-sectional observational study was held. This study was conducted to assess the hypothesis that a higher prevalence of hypovitaminosis D exists in the elderly population with a hip fracture as compared with patients with knee/hip osteoarthritis or lumbar spondylosis in a south-western Mediterranean region.

Methods

This study included 61 consecutive patients with a mean age of 83 years who sustained a hip fracture (Group A). Sixty patients, with an average age of 73 years, who suffered from degenerative hip/knee osteoarthritis or lumbar spondylosis were subsequently selected as the control group (Group B). Parathyroid hormone (PTH) and 25-hydroxy VD blood levels were measured.

Results

Ninety-six point seven percent (96.7%; 59/61) of the individuals with hip fracture and 81.7% (49/60) in the controls were found with abnormal VD values (<30 ng/ml). The comparison of vitamin D values between the total samples of group A and B revealed a statistically significant difference (unpaired t-test, p<0.0001) while both male (p=0.0049) and female (p<0.0001) individuals in group A also showed statistically significant lower VD levels than their counterparts did. In addition, increased levels of parathormone were observed in women of group A (p=0.0016) and, therefore, for group A in the total sample (p=0.0004) while no statistical significance was observed in males (p=0.7712). Age was found to be an independent risk factor for VD deficiency in both groups (Group A p=0.04, Group B p=0.043). It is noteworthy that only four patients from group B (6, 67%) and none from group A had undergone blood tests for VD and PTH evaluation before hospital admission.

Conclusions

Τhe results confirmed the initial hypothesis of the study. Although VD hypovitaminosis concerns the majority of elderly living in this south-western Mediterranean region, the authors suggest VD and PTH measurements regardless of annual insolation, to identify and counsel the elderly with an increased risk of hip fracture and to avoid perioperative complications in patients who undergo elective orthopedic surgeries.

## Introduction

Ιn the elderly, osteoporosis is an important public health issue, as it is directly related to fragility fractures [1] and associated complications. Hip fracture (peritrochanteric, femoral neck fracture) is a major consequence of senile osteoporosis, which may lead to significant morbidity and mortality and, simultaneously, to a dramatic increase in health and social costs [2].

Vitamin D (VD) deficiency seems to be an underestimated cause of osteoporosis [3], especially in countries with a significant duration of sunlight throughout the year, as in this Mediterranean country where this study was held. However, chronic low calcium deficiency due to low VD level uptake causes the accumulation of non-calcified osteoid tissue in long bones and the spine, making these particular bones fragile [4]. In addition, VD deficiency is considered an additional risk factor for falls while adequate calcium and vitamin D uptake seems to reduce significantly the risk of new falls [5] and, consequently, new hip fractures.

It is considered that VD deficiency causes sarcopenia, or muscle weakness, while it contributes to an increased risk for falls [6]. Although there is no commonly accepted definition of VD deficiency, its range varies between 10 and 30 ng/ml. According to Holick’s criteria [7], the lowermost limits for different VD values are: <10 ng/ml (severe deficiency), <20 ng/ml (deficiency), 21-29 ng/ml (inadequacy), and > 30 ng/ml for vitamin D (adequacy). Based on Holick’s criteria [7], most studies recommend 30 ng/mL as the lower limit of normal and desirable levels of 25-hydroxyvitamin VD.

The prevalence of 25-hydroxy VD inadequacy appears to be higher in patients with hip fractures [8] while severe VD deficiency seems to be associated with osteoporotic hip fractures of comparable severity [9]. One of the goals of this study was to identify the characteristics of each gender to distinguish the elderly with an increased risk for hip fracture. Thus, elderly individuals, with increased risk for hip fracture, could be targeted for having all the appropriate interventions for fracture prevention such as correction of VD deficiency, bone density assessment (DEXA), falls prevention counseling, medication review, vision and hearing evaluation, walking disorders correction, home environment modification, as well as encouragement for daily mild exercise.

On the other hand, while the joint (knee, hip) arthroplasties frequency rises, recent literature highlights that preoperative VD deficiency is associated with higher postoperative complication rates [10]. Furthermore, osteoporosis and osteoarthritis commonly coexist in the elderly [11] while VD deficiency contributes also to bone fragility. Secondary hyperparathyroidism has been suggested as the main mechanism due to VD deficiency and, consequently, this condition contributes to the pathogenesis of secondary osteoporosis. However, bone density, VD, and parathyroid hormone levels are rarely measured before elective surgeries (hip, knee replacement), so several disastrous complications can arise [12].

This prospective study was conducted to assess the hypothesis that a higher prevalence of hypovitaminosis D exists in the elderly population with a hip fracture as compared with patients with knee/hip osteoarthritis or lumbar spondylosis in a south-western Mediterranean region.

## Materials and methods

Patients ≥ 65 years with a hip fracture or musculoskeletal degenerative disease (knee/hip arthritis or lumbar spondylosis) admitted to the orthopedic department of “Agios Andreas” General Hospital of Patras, Greece from January 2018 to January 2019, were screened on the basis of the age, disease, and exclusion criteria to be enrolled in the study. Patients with a history of major trauma (e.g. traffic accident, fall from height), vitamin D supplementation, body mass index ≥28, residents of geriatric institutions, and medical history of chronic conditions affecting bone mineral density (e.g. corticosteroid uptake, Paget disease) were excluded. Furthermore, patients with increased PTH and impaired calcium levels were excluded from the study and referred to the endocrinology department for further investigation.

As the control group, consecutive elderly patients aged ≥ 65 years with knee/hip osteoarthritis or lumbar spondylosis were selected to match in age to the patients with hip fractures.

One-hundred twenty-one patients fulfilled the participation eligibility criteria. The group with the hip fracture consisted of 61 patients (Group A) while in the group with the degenerative disease 60 patients were inserted (Group B).

Ethical approval was obtained from the ethics committee of the “Agios Andreas” General Hospital of Patras and informed written consent was obtained from all the participants. Subjects could withdraw from the study at any time, either based on their personal decision or at the discretion of the researcher.

The total serum concentration of 25-hydroxy VD (25OH Vit D) and parathyroid hormone (PTH) were measured by automated enzymatic immunoassay. Serum VD levels were classified as follows: severe deficiency (<10 ng/mL), mild vitamin D deficiency (<20 ng/mL), vitamin D inadequacy (from 20 to 29 ng/mL), and normal vitamin D levels (adequacy > 30 ng/mL) according to Holick's classification. A PTH level of 65 pg/ml or more was considered as the threshold above which the values were indicative of hyperparathyroidism, which in combination with low VD levels and normal calcium values was considered secondary.

Statistical analysis was performed using the SPSS statistical package, version 20.00 (IBM Corp, Armonk, NY). P-value <0.05 was set as the level of statistically significant difference. Results were expressed as mean values and standard deviations in parametric variables or as a percentage in non-parametric variables. The differences between different parametric variables assessed using the unpaired t-test. The chi-square test was used for non-parametric variable comparisons. Different parametric variables were compared with Pearson's equation while nonparametric and parametric variables were compared with Spearman's equation.

## Results

In group A, we included 51 women and 10 men; 44 patients suffered from peritrochanteric fractures and 17 from femoral neck fractures (Table 1).

**Table 1:** Hip fracture type in group A sorted by gender.

Hip fracture type	Male		Female		p-value
	Peritrochanteric fracture	Femoral neck fracture	Peritrochanteric fracture	Femoral neck fracture	0.091
	9	1	35	16	

Group B was composed of 60 patients: 38 women and 22 men, who suffered either from knee osteoarthritis (Knee OA): 25 patients; or hip osteoarthritis (Hip OA): 22 patients; and 13 with degenerative spinal lumbar stenosis (DSLS). The correlation between gender and the type of degenerative disease revealed no statistical significance (chi-squared test, p=0.42) (Table 2).

**Table 2:** Type of degenerative disease in group B sorted by gender OA, osteoarthritis; DSLS, degenerative spinal lumbar stenosis

Degenerative disease	Male					Female					p-value
	Knee OA	Hip OA		DSLS		Knee OA	Hip OA		DSLS		0.412
	8		8		6	17		14		7	

Sixty-nine percent (69%) of the female patients in group A had a peritrochanteric fracture and 31% a femoral neck fracture. The vast majority of men in group A (90%) suffered from a peritrochanteric fracture and only 10% from a femoral neck fracture (Table 1). No statistically significant differences were found between gender and type of fracture in group A (chi-squared test, p=0.091) (Table 1).

The average age was 83 (range 65-93 years) in group A patients and 73 (range 65-87) years in group B. No statistically significant difference was found in the age of patients of the two groups (unpaired t-test, p= 0.429). No statistical significance was found either comparing the age of male subjects of two groups (p=0.607) or the females (p=0.304) (Table 3).

**Table 3:** Presentation of patient’s age sorted by gender and group Group A, patients with hip fracture; Group B, patients with musculoskeletal degenerative disease (knee/hip arthritis or lumbar spondylosis)

Age	Male					Female					Total Sample				
	Group A		Group B		p-value	Group A		Group B		p-value	Group A		Group B		p-value
	80.60±7.64	73.59±7.53		0.607		83.94±6.31	72.63±6.15		0.304		83.39± 6.59	72.98± 6.65		0.429	

Vitamin D and PTH levels are summarized and sorted by gender and group. The comparison of vitamin D values between the total sample of group A and B revealed a statistically significant difference (unpaired t-test, p<0.0001), while both male (unpaired t-test, p=0.0049) and female (unpaired t-test, p<0.0001) individuals in group A also showed statistically significant lower VD levels than their counterparts. In addition, increased levels of parathormone were observed in women of group A (unpaired t-test, p=0.0016) and therefore for group A in the total sample (unpaired t-test, p=0.0004) while no statistical significance was observed in males (unpaired t-test, p=0.7712; Table 4).

**Table 4:** Cumulative presentation of vitamin D and PTH levels sorted by gender and group Vit-D, vitamin d; PTH, parathyroid hormone; Group A, patients with hip fracture; Group B, patients with musculoskeletal degenerative disease (knee/hip arthritis or lumbar spondylosis)

Vit-D (ng/ml)	Male			Female			Total		
	Group A	Group B	p-value	Group A	Group B	p-value	Group A	Group B	p-value
	10.39±8.39	22.51±11.23	0.0049	8.75±7.56	18.68±9.15	<0.0001	9.02±7.65	20.08±10.04	<0.0001
PTH (pg/ml)	36.13±11,19	34.48±16.03	0.7712	79.37±82.52	34.93±17.75	0.0016	72.28±77.16	34.77±17.00	0.0004

In order to highlight the incidence of VD deficiency in both groups, we present the categorization of both groups to the particular status of VD, according to the Holick classification, sorted by numbered cases and as percentages. Sixty-nine percent (69%) of group A patients had a severe vitamin D deficiency (1-10 ng/ml), whereas vitamin D efficiency (>30 ng/ml) was found only in 18.3% of group B patients (Table 5).

**Table 5:** Cumulative presentation of vitamin D status sorted by group Status Vit-D, status of vitamin D; Group A, patients with hip fracture; Group B, patients with musculoskeletal degenerative disease (knee/ hip arthritis or lumbar spondylosis)

Status of Vit-D	Group A		Group B		Total	
	Cases(N)	(%)	Cases	%	Cases	%
Severe deficiency (1-10 ng/ml)	42	68.9	11	18.3	53	43.8
Deficiency (10-20 ng/ml)	13	21.3	23	38.3	36	29.7
Inadequacy (20-30 ng/ml)	4	6.6	15	24.6	19	15.8
Adequacy (>30 ng/ml)	2	3.3	11	18.3	13	10.7
	61	100	60	100	121	100

Finally, age was found to be the only independent risk factor for VD deficiency in both patient groups. In group A, age was correlated with VD levels (Pearson r=0.260, p=0.043) and in group B (Pearson r=0.364, p=0.04).

It is noteworthy that only four patients (6.67%) from group B and none from group A underwent blood tests for vitamin D and parathyroid hormone evaluation before hospital admission.

## Discussion

The literature concerning the hypovitaminosis D (<25 ng/ml) varies a lot. Thus, vitamin D deficiency was found in 65% of patients with a hip fracture in Russia, 67% in Italy, 57.5% in Egypt, 76.7 in Singapore, and 90% in Japan [13]. Surprising, our results are even worse than those previously reported in other countries since VD deficiency with the definition of 25 ng/ml was present in 95.1% of those aged> 65 years with a hip fracture and in 63.3% of the elderly population with degenerative disease. According to Holick’s definition, 96.7% of hip fracture patients had low vitamin D levels (<30 ng/ml) while the prevalence of hypovitaminosis D among the elderly with degenerative diseases was 81.7%. An increased incidence of hip fractures in the female population was also found in this study (85%), and it was also observed in other western countries [14].

Our study confirms our hypothesis that elderly patients with hip fractures (Group A) are associated more frequently with vitamin D deficiency in comparison with elderly people with osteoarthritis (p<0.0001). It should be taken into consideration that the average age of Group A is about 10 years more, and there are 13 more women in this group. However, the unexpected result derived from our study was the high incidence of hypovitaminosis D among the elderly without a hip fracture in a sunny Mediterranean European country. Sunlight is the best way of VD synthesis. When VD is produced in the skin, 100% of it is potentially bound to the VD binding protein (VDR) while when vitamin D3 is ingested from the diet or supplements, approximately only 60% of the vitamin D3 is bound to VDR. Aging, skin pigmentation, obesity, and increased sun protection are aggravating factors for adequate sunlight vitamin D production and should be recognized. Moreover, the prevalence of vitamin D deficiency in patients with osteoarthritis or spondylosis in our study was similar or higher than the reported values of studies carried out in northern European countries such as Finland, Germany, and the UK [15], where annual insolation is significantly lower. However, in these countries, the consumption of VD-enriched foods is widespread.

Furthermore, a literature review revealed interesting data from a study of northern Greece, in which authors found out 96.9% of the deficiency or insufficiency of serum levels of 25-hydroxyvitamin D in patients with knee or hip osteoarthritis [16]. In daily clinical practice, VD, PTH, and bone density are very rarely evaluated in patients with hip or knee osteoarthritis and lumbar degenerative spinal stenosis scheduled for surgical intervention. Surgeons who perform the above-mentioned surgical interventions declare that reduced bone density and affected local bone quality will influence their choice of implant selection both for arthroplasties as well as spinal fusion, as recorded in recent studies [12,17].

In addition, there is evidence that preoperative vitamin D deficiency is associated with higher postoperative complication rates in patients who undergo knee arthroplasty [10] because VD has various effects and does not affect only the bone density. Indeed, thousands of cellular and animal studies indicate that VD signaling has a profound effect on most physiologic processes, including cancer prevention, improved cardiovascular function, diabetes prevention, prevention of obesity, improved muscle function, enhanced barrier function of the skin, hair follicle cycling, and prevention of immune-related diseases [18].

VD deficiency is also associated with poor postoperative functional outcomes both in patients who undergo total hip [19] and knee [11] arthroplasty. Elderly patients over 65 years, who undergo total hip and knee arthroplasty, as well as spine fusion, are very likely to have low levels of VD, which can cause secondary hyperparathyroidism. Parathyroid hormone-induced osteoclastogenesis induces enzymatic degradation of bone matrix and induces the secretion of hydrochloric acid, calcium, and phosphorus. The consequences of the above processes are poor bone mineralization, reduced bone density, osteoporosis, reduced bone strength, and increased potential for fragility fractures [20]. This condition, accompanied with type 1 and type 2 osteoporosis, results in significant bone mass loss. Low bone density is known to affect the primary stability of orthopedic implants [21] and very often leads to complications such as loosening [11], periprosthetic fractures, and additional postoperative complications [10]. Unfortunately, this issue has not received appropriate attention from physicians, and only 4% of patients undergoing these procedures perform bone density measurement preoperatively [12] while a much smaller proportion undergoing the laboratory testing of VD and parathyroid hormone. Surgeons who perform joint arthroplasties or lumbar fusions should be aware of the high prevalence of VD deficiency in patients with degenerative disease. The role of vitamin D supplementation in the treatment or prevention of OA remains uncertain. A systematic review of randomized controlled trials (RCTs) concludes that evidence from RCTs is insufficient to support the use of VD supplementation for patients with knee osteoarthritis [22]. More research is needed to reconcile these conflicting findings, although the literature clearly supports preoperative vitamin D supplementation in cases of insufficiency, to minimize complications [23].

As seen in the results presented above, women were found to have lower levels of VD in both groups of patients, indicating an increased incidence of hypovitaminosis D in the female sex. Many studies have confirmed this fact [24], as women have a gradual decline in postmenopausal VD levels and aging intensifies the hypovitaminosis. A large study with a 90,000-women sample revealed an increased incidence of hypovitaminosis D and increased risk for hip fracture [25]. For these reasons, it is recommended to evaluate VD both in menopausal and older women.

Despite the increased incidence of hypovitaminosis D in women, men with a hip fracture showed a remarkable, statistically significant deficiency of vitamin D levels in the present study in comparison with their counterparts with osteoarthritis. The clinical application of this result that is drawn is that older men who have low vitamin D levels are at increased risk of hip fracture. This finding has also been formulated in another large study, which included 1,608 elderly men and concluded that men who had low levels of vitamin D also had an increased risk of proximal femoral fracture [26] while another study revealed that elderly men with VD deficiency present increased possibilities for hip fracture and bone mineral density reduction [27].

The parathyroid hormone plays a key role in calcium metabolism in the body, as it stimulates bone resorption, increases serum calcium and phosphorus levels, and promotes the synthesis of 1,25 (OH) 2D3. The main regulator for PTH secretion is the receptor sensitive to changes in calcium concentration (CaSR = Calcium Sensitive Receptor). Thus, CaSR activation by calcium inhibits PTH synthesis rapidly. Parathyroid glands also express high levels of VD receptors (VDR), which are activated by the binding of 1,25 (OH) 2D3 and reduce the synthesis of PTH. In contrast, VD deficiency stimulates the synthesis of PTH [28].

In the present study, we found a statistical significance in the parathormone levels among elderly women with hip fractures (group A) and therefore for group A in the total sample while no statistical significance was observed in males. The clinical application of this result that is drawn is that older women who have both low vitamin D levels and increased levels of parathormone are at a greater risk of hip fracture.

It is worth noting that age was found to be the only independent risk factor for VD deficiency in both groups of elderly patients. These findings are in line with the current literature, as a recent study of 125 patients supports that age is an independent risk factor for hypovitaminosis D in older patients [29]. Based on the literature and our study, there is evidence that as elderly people get older, VD deficiency increases. However, inadequate VD levels are a common condition among the elderly irrespective of their age and, therefore, all seniors should be investigated for possible VD deficiency and treated appropriately. In recent studies, the correlation between VD deficiency and age was significant only in patients over 70 years of age [13] while other studies found a significant association between bone loss and low 25 (OH) D levels in subjects over the age of 75 years old [30].

A limitation of this study is the relatively small number of patients and thus moderate power to detect significant effects. Although, to our knowledge, there is no study in the literature to compare VD levels among the elderly with a hip fracture and knee/hip osteoarthritis or lumbar spondylosis. Furthermore, the search of the literature didn’t reveal any result concerning VD levels among the elderly with a hip fracture in sunny Greece. Another possible limitation of our study is the unequal distribution of men and women in both groups while there is no complete correlation of the mean age of the subjects between the two groups; however, the comparison of patient’s age between the two groups wasn’t found to be statistically significant. Furthermore, unfortunately at our center, we cannot measure the bone mineral density of our patients using the dual-energy photon absorption method, as we don’t have the necessary equipment. This parameter would significantly help us correlate vitamin D levels with bone density levels, confirming the cases of affected bone mass. More and larger studies from sunshine regions and Mediterranean countries are definitively required for further evaluation of this increasing public health issue.

## Conclusions

Our study confirmed that elderly patients with hip fractures in Greece have less vitamin D adequacy and increased parathormone levels in comparison with elderly people with osteoarthritis. However, the elderly with osteoarthritis have significantly reduced levels of vitamin D. Recent studies correlate hypovitaminosis D with an increased risk of adverse complications in patients undergoing arthroplasties. This finding, accompanied by hypovitaminosis D-induced osteoporosis, should alert surgeons performing such interventions. Moreover, the study demonstrated that age constitutes an independent aggravating factor for hypovitaminosis D among the elderly either with a hip fracture or osteoarthritis, and this finding should be taken also under consideration by physicians. Αs VD hypovitaminosis concerns the majority of elderly living in this south-western Mediterranean region, the authors suggest VD and PTH measurements, regardless of annual insolation, to identify and counsel the elderly with an increased risk of hip fracture and to avoid perioperative complications in patients who undergo elective orthopedic surgeries.
